# Prognostic values of negative estrogen or progesterone receptor expression in patients with luminal B HER2-negative breast cancer

**DOI:** 10.1186/s12957-016-0999-x

**Published:** 2016-09-13

**Authors:** Chansub Park, Kyeongmee Park, Jiyoung Kim, Youngjoo Sin, Inseok Park, Hyunjin Cho, Keunho Yang, Byung Noe Bae, Ki Whan Kim, Sookyung Ahn, Geumhee Gwak

**Affiliations:** 1 Department of Surgery, Sanggye Paik Hospital, College of Medicine, Inje University, 1342 Dongil-ro, Nowon-gu, Seoul, 139-707 Korea; 2 Department of Pathology, Sanggye Paik Hospital, College of Medicine, Inje University, Seoul, Korea; 3 Department of Diagnostic Radiology, Sanggye Paik Hospital, College of Medicine, Inje University, Seoul, Korea; 4 Department of Radiologic Oncologist, Sanggye Paik Hospital, College of Medicine, Inje University, Seoul, Korea; 5 Department of Surgery, Kangnam Sacred Heart Hospital, College of Medicine, Hallym University, Seoul, Korea

**Keywords:** Breast cancer, Estrogen receptor, Luminal B, Progesterone receptor, Prognosis

## Abstract

**Background:**

The luminal subtype of breast cancer is sensitive to anti-estrogen therapy and shows a better prognosis than that of human epidermal growth factor receptor2 (HER2)-enriched or triple-negative breast cancer. However, the luminal type of breast cancer is heterogeneous and can have aggressive clinical features. We investigated the clinical implications of single hormone receptor negativity in a luminal B HER2-negative group.

**Methods:**

We collected luminal B HER2-negative breast cancer data that were estrogen receptor (ER) and/or progesterone receptor (PR) positive, Ki 67 high (>14 %), and HER2 negative and divided them into the ER- and PR-positive group and the ER- or PR-negative group. We analyzed the clinical and pathological data and survival according to ER or PR loss.

**Results:**

There were no statistical differences in TNM stage, breast and axillary operative methods, or number of tumors between the ER- and PR-positive group and ER- or PR-negative group. However, the ER- or PR-negative group was associated with older age (≥45 years), higher histological grade, lower Bcl-2 expression, and far higher Ki 67 (>50 %). Disease-free survival (DFS) and overall survival (OS) were shorter in the ER- or PR-negative group than that in the ER- and PR-positive group (*p* = 0.0038, *p* = 0.0071).

**Conclusions:**

ER- or PR-negative subgroup showed worse prognosis than ER- and PR-positive subgroup in the luminal B HER2-negative group. We could consider the negativity of ER or PR as prognostic marker in luminal B HER2-negative subtype of breast cancer.

**Electronic supplementary material:**

The online version of this article (doi:10.1186/s12957-016-0999-x) contains supplementary material, which is available to authorized users.

## Background

Gene expression profiling studies for breast cancer have unveiled that breast cancer has very heterogeneous biological characteristics and at least four molecular distinct subtypes, such as luminal A and B, human epidermal growth factor receptor2 (HER2)-enriched, and basal-like subtypes have been identified over the few last decades [1–3]. Personalized therapy according to gene expression profiles for breast cancer patient has been possible to achieve optimal therapeutic effects [4–6]. Many investigators have been making a more progresses for specific clinical conditions in breast cancer patients, such as inflammatory breast cancer, recurrent or metastatic breast cancer, hereditary breast cancer, and basal-like breast cancer [7–11].

The estrogen receptor (ER) and progesterone receptor (PR) are traditional prognostic and predictive factors in breast cancer, and both of them are the mainstays of gene expression profiles to determine intrinsic breast cancer subtypes. ER- or PR-positive breast cancers are classified as the luminal subtype, which have a more favorable prognosis and are more responsive to anti-estrogen therapy than that of ER- and PR-negative breast cancer [12–14]. However, the luminal subtype of breast cancer is very heterogeneous and occasionally has very aggressive clinical features. Luminal type breast cancer has two biologically distinct subtypes, luminal A and luminal B, and it is well known that luminal B subtype have higher proliferative characteristics and poorer prognosis than those of luminal A [15].

A number of studies have been reported that luminal B subtype is dramatically distinct from luminal A subtype at the cellular signaling pathway and DNA levels, including growth factor receptors, such as epidermal growth factor receptor (EGFR; HER1) and HER2 as well as its downstream signaling pathway [16]. The St. Gallen International Expert Consensus on the Primary Therapy of Early Breast Cancer 2013 distinguished luminal A-like breast cancer from luminal B-like disease based on immunohistochemical stains of ER, PR, and Ki-67 status without a requirement for molecular diagnostics. Recently, we have figured out the heterogeneity of luminal B HER2-negative group from our consecutive clinical studies, and we wanted to find out which factors affect the disease prognosis that probably related with disease heterogeneity within luminal B subtype. We performed this study to determine the clinical implications of single hormone receptor loss by immunohistochemical (IHC) staining methods in a luminal B HER2-negative group.

## Methods

We collected clinical and pathological data from patients with breast cancer who underwent breast surgery and treatment between January 2004 and December 2014 at Sanggye Paik Hospital. Among the 769 patients, we selected 183 with luminal B HER2-negative breast cancers that were ER or PR positive, Ki-67 > 14 %, and HER2 negative. We divided them into two groups of the ER and PR positive group and the ER or PR negative group. ER and PR positive group defined as both hormonal receptor showed positive reaction. These two group was compared disease free survival (DFS) and overall survival (OS) between the two groups. We also analyzed the clinical and pathological data of each group, including age, breast and axilla operative methods, tumor type, TNM stage, histological grade, nuclear grade, number of tumors, Ki-67 and Bcl-2 expression, and recurred or metastatic sites. We choose median 45 age, for statistical convenience.

### IHC staining for ER, PR, Bcl-2, and Ki 67

The ER NCL-1-ER-6F11 and PR NCL-L-PGR-312 liquid mouse monoclonal antibodies (Leica Microsystems Inc., Newcastle Upon Tyne, UK) diluted 1:80 with normal goat serum (diluted 1:5 TBS) were used as the primary antibodies for the ER and PR assays, respectively. The secondary antibody was goat anti-mouse peroxidase conjugated immunoglobulin, and 3,3′-diaminobenzidine tetrahydrochloride (DAB) was used as the chromogen. We scored ER and PR as 0, 1+, 2+, and 3+ according to staining intensity with a description of the percentage related to the proportion of stained nuclei in 10 high power fields [17]. We defined ER and PR positivity as any positive score or a percentage greater than zero. We converted the intensity scores and proportion percentages into the Allred score [18]. We determined Allred score 0, 2 is negative, and 3 to 8 is positive [19].

We performed IHC for Bcl-2 and Ki-67 using the avidin-biotin peroxidase complex method with aminoethylcarbazole as the chromogen and the Vectastain ABC Elite kit (Vector Laboratories, Burlingame, CA, USA). We counterstained the sections with Mayer’s hematoxylin. Sections was incubated in monoclonal mouse anti-human Bcl-2 oncoprotein to assess Bcl-2 (1:100; Dako, Glostrup, Denmark), and brown nuclear immunostaining was examined. Sections were incubated with monoclonal mouse anti-human Ki-67 antigen for the Ki 67 measurements (1:100; Dako), and brown nuclear immunostaining was examined [17]. We defined Bcl-2 overexpression as Bcl-2 intensity over 10 %. In addition, low PR expression was defined as Allred score 3, 4. In Ki-67, we choose cutoff value at 50 %. According to modified 2013 St. Gallen Consensus, high Ki-67 defined 20 % cutoff. Nevertheless, we wanted to know higher Ki67 labeling index might have association with ER or PR expression.

### IHC staining for HER2/*neu*

We performed IHC of the HER2/neu protein on 4-μm-thick paraffin embedded tissue sections on poly-l-lysine-coated slides. After deparaffinization and blocking of endogenous peroxidase, we performed HER2/neu immunostaining using the rabbit anti-human c-erbB-2 oncoprotein as the primary antibody (Dako) at a 1:100 dilution. Binding of the primary antibody was detected using the Dako Quick-Staining, labeled streptavidin-biotin system (Dako, Carpentaria, CA, USA), followed by adding the DAB chromogen. Two pathologists scored each slide according to the manufacturer’s recommended criteria in a blinded fashion. We red immunostaining in a semi-quantitative manner and graded as follows: 0, 1+, 2+, and 3+. We designated intensity scores of 0 or 1+ as negative expression and 3+ as positive expression for HER2/neu. We considered a 2+ score as equivocal, which was subjected to silver-enhanced in situ hybridization (SISH) analysis [17].

### SISH for HER2

We performed HER2 SISH on the Ventana Benchmark automated instrument (Ventana Medical Systems, Tucson, AZ, USA), according to the manufacturer’s protocols for INFORM HER2 DNA and chromosome 17 probes. We performed testing for the HER2 gene and chromosome 17 on sequential sections. Two sections were baked at 60 °C for 20 min. The HER2 DNA probe was denatured at 95 °C for 12 min, and hybridization was performed at 52 °C for 2 h. The chromosome 17 probe was denatured at 95 °C for 12 min, and hybridization was performed at 44 °C for 2 h. After hybridization, appropriate stringency washes were performed three times at 72 °C for the HER2 probe and three times at 59 °C for the chromosome 17 probe. Both DNP-labeled probes were visualized using a rabbit anti-DNP primary antibody and the ultraView SISH Detection Kit (Ventana). The slides were counterstained with hematoxylin for examination by light microscopy. Evaluation of HER2 gene amplification status was performed in a blinded manner using the ASCO/CAP guidelines [20].

### Statistical methods

We used the chi-square test to analyze the clinic-pathologic factors affecting prognosis between the ER- or PR-negative group and the ER- and PR-positive group. We analyzed the difference in DFS between the ER- or PR-negative and the ER- and PR-positive groups by the Kaplan–Meier method. A univariate analysis of the clinic-pathologic factors affecting prognosis in both groups was conducted with the log-rank test. We used the Cox multivariate regression model for the multivariate analysis. MedCalc Statistical Software ver. 15.5 software (MedCalc Software, Ostend, Belgium; https://www.medcalc.org; 2015) was used for the statistical analysis. We considered a *p* value <0.05 as statistically significant value.

## Results

We collected 184 luminal B HER2-negative breast cancers from 769 patients with breast cancer. Among them, ER negative observed in four patients and PR negative in 20 patients. Thus, the ER- and PR-positive group included 160 cases and the ER- or PR-negative group had 24. PR-negative group included 20 cases, PR low group had 44, and PR high group had 120.

### Univariate and multivariate analyses of the pathologic characteristics according to ER or PR status

The median age of the patients was 48.5 years in the ER- and PR-positive group and 55.5 years in the ER- or PR-negative group. A significant difference between the ER- and PR-positive and ER- or PR-negative groups was detected when we used 45 years as the age cutoff value (*p* = 0.0336). Fifty-five patients (29.9 %) were TNM stage I, 92 (50.0 %) were stage II, 33 (17.9 %) were stage III, and 4 (2.2 %) were stage IV. In total, 129 patients (70.1 %) received breast-conserving surgery and 55 received mastectomy. Axillary lymph node dissection was performed in 112 patients (60.9 %), sentinel lymph node biopsy in 70 patients (38.0 %), and an axillary procedure was omitted in 2 patients. No significant differences in TNM stage, operative method, axillary lymph node evaluation method, or number of tumors were observed between the ER- and PR-positive and ER- or PR-negative groups (Table 1).

**Table 1:** The clinic-pathologic characteristics of luminal B HER2-negative breast cancer

	ER and PR positive *N* = 160 (%)	ER or PR negative *N* = 24 (%)	*p* value
Age (years) median	48.5	55.5	0.0336
= or <45	59 (36.9)	3 (12.5)	
>45	101 (62.1)	21 (87.5)	
TNM stage (AJCC 8th)			0.1061
I	50 (31.2)	5 (20.8)	
II	81 (50.6)	11 (45.8)	
III	27 (16.9)	6 (25.0)	
IV	2 (1.3)	2 (8.3)	
Breast operation			0.8761
BCS	113 (70.6)	16 (66.7)	
Mastectomy	47 (29.4)	8 (33.3)	
Axillary node operation			0.2088
SLNB	63 (39.4)	7 (29.2)	
ALND	96 (60.0)	16 (66.7)	
omit	1 (0.6)	1 (4.2)	
Histologic type			0.1587
Invasive ductal carcinoma	142 (88.8)	22 (91.7)	
Invasive Lobular carcinoma	5 (3.1)	2 (8.3)	
Mucinous carcinoma	6 (3.8)	0 (0)	
Papillary carcinoma	6 (3.8)	0 (0)	
Micropapillary carcinoma	3 (1.9)	0 (0)	
Apocrine carcinoma	1 (0.3)	0 (0)	
Histologic grade			0.4421
II	31 (19.4)	8 (33.3)	
III	101 (63.1)	16 (66.7)	
Not available	28 (17.5)	0 (0)	
Nuclear grade			0.9403
I	82 (51.2)	14 (58.3)	
II	45 (28.1)	9 (37.5)	
III	5 (3.1)	1 (4.2)	
Not available	28 (17.5)	0 (0)	
Number of tumor			0.6269
Single	137 (85.6)	22 (91.7)	
Multiple	23 (14.4)	2 (8.3)	
Bcl-2 expression			0.0006
= or <10 %	17 (10.9)	11 (39.3)	
>10 %	139 (89.1)	17 (60.7)	
Ki 67			0.0167
= or <50 %	136 (85.0)	15 (62.5)	
>50 %	24 (15.0)	9 (37.5)	
Adjuvant therapy			
Chemotherapy			
Yes	133 (83.1)	22 (91.7)	0.1321
Anthracycline/taxane based	72 (45.0)	17 (70.8)	
CMF or CNF based	50 (36.9)	5 (20.7)	
No	27 (16.9)	2 (8.3)	
Hormonal therapy			
Yes	148 (92.5)	22 (91.7)	0.0579
SERM	100 (62.5)	9 (37.5)	
AI	49 (30.6)	13 (54.2)	
No	11 (6.9)	2 (8.3)	
Radiation therapy			
Yes	110 (68.7)	17 (70.8)	0.9752
No	50 (31.2)	7 (29.2)	

*TNM* tumor size/node/metastasis, *AJCC* American Joint Committee on Cancer, *BCS* breast conserving surgery, *SLNB* sentinel lymph node biopsy, *ALND* axillary lymph node dissection, *SERM* selective estrogen receptor modulator, *AI* aromatase inhibitor, *CMF* cyclophosphamide/methotrexate/5-fluorouracil, *CNF* cyclophosphamide/vinorelbine/5-fluorouracil

However, the ER- or PR-negative group was significantly correlated with a lower Bcl-2 (≤10 %) expression (*p* = 0.0006) and a far higher Ki-67 index (>50 %) (*p* = 0.0167) than those in the ER- and PR-positive group (Table 1). In terms of relapse, bone and liver metastases were significantly more frequent in the ER- or PR-negative group than those in the ER- and PR-positive group (*p* = 0.0297, *p* = 0.0093, respectively) (Table 2). The multivariate analysis revealed that Bcl-2 expression (*p* = 0.0012) was significantly related with ER or PR negative, respectively (Table 3).

**Table 2:** The analysis of recurrence pattern of luminal B HER2-negative breast cancer

Recurrence site	ER and PR positive *N* = 160 (%)	ER or PR negative *N* = 24 (%)	*p* value
Breast			0.7407
No	153 (95.6)	22 (91.6)	
Yes	7 (4.4)	2 (8.4)	
Bone			0.0297
No	147 (91.9)	18 (75.0)	
Yes	13 (8.1)	6 (25.0)	
Lung			0.0873
No	152 (95.0)	20 (83.3)	
Yes	8 (5.0)	4 (16.7)	
Liver			0.0093
No	153 (95.6)	19 (79.2)	
Yes	7 (4.4)	5 (20.8)	
Brain			0.8510
No	158 (98.7)	23 (95.8)	
Yes	2 (1.3)	1 (4.2)	
Lymph node			0.2483
Yes	153 (95.6)	21 (87.5)	
No	7 (4.4)	3 (12.5)

**Table 3:** Multivariate analysis of factors associated with ER or PR negativity

Variable	Coefficient	Std. error	Odds ratio	95 % CI	*p* value
Age (median 45)	−0.64939	0.50740	0.5224	0.1932 to 1.4121	0.2006
Bcl-2 (cutoff 10 %)	1.54314	0.47871	4.6793	1.8310 to 11.9580	0.0012
Ki 67 (cutoff 50 %)	−0.70809	0.49583	0.4926	0.1864 to 1.3018	0.1533

*CI* confidence interval

### Adjuvant therapies in luminal B HER2-negative breast cancer

Adjuvant systemic chemotherapy was performed for 133 of the 160 patients in the ER- and PR-positive group (83.1 %) and for 22 of the 24 patients in the ER- or PR-negative group (91.7 %) The chemotherapy regimens were anthracycline and/or taxane-based, cyclophosphamide/methotrexate/5-fluorouracil, and cyclophosphamide/vinorelbine/5-fluorouracil.

Adjuvant hormonal therapy was performed for 148 of the 160 patients in the ER- and PR-positive group (92.5 %) and for 22 of the 24 patients in the ER- or PR-negative group (91.7 %). A slight difference in the choice of drugs was observed between the two groups. The selective estrogen receptor modulators (SERM), such as tamoxifen and toremifene, were prescribed more frequently in the ER and PR positive than that in the ER- or PR-negative group (62.5 vs. 37.5 %). In addition, aromatase inhibitors, such as anastrozole and letrozole, were prescribed more frequently in the ER- or PR-negative group than those in the ER- and PR-positive group. (54.2 vs. 30.6 %) (*p* = 0.0579).

In total, 110 (68.7 %) of the 160 patients in the ER- and PR-positive group, and 17 (70.8 %) of the 24 in the ER- or PR-negative group received radiation therapy for breast and/or regional axillary lymph nodes (Table 1).

### DFS and OS based on ER or PR status

**Fig. 1:**
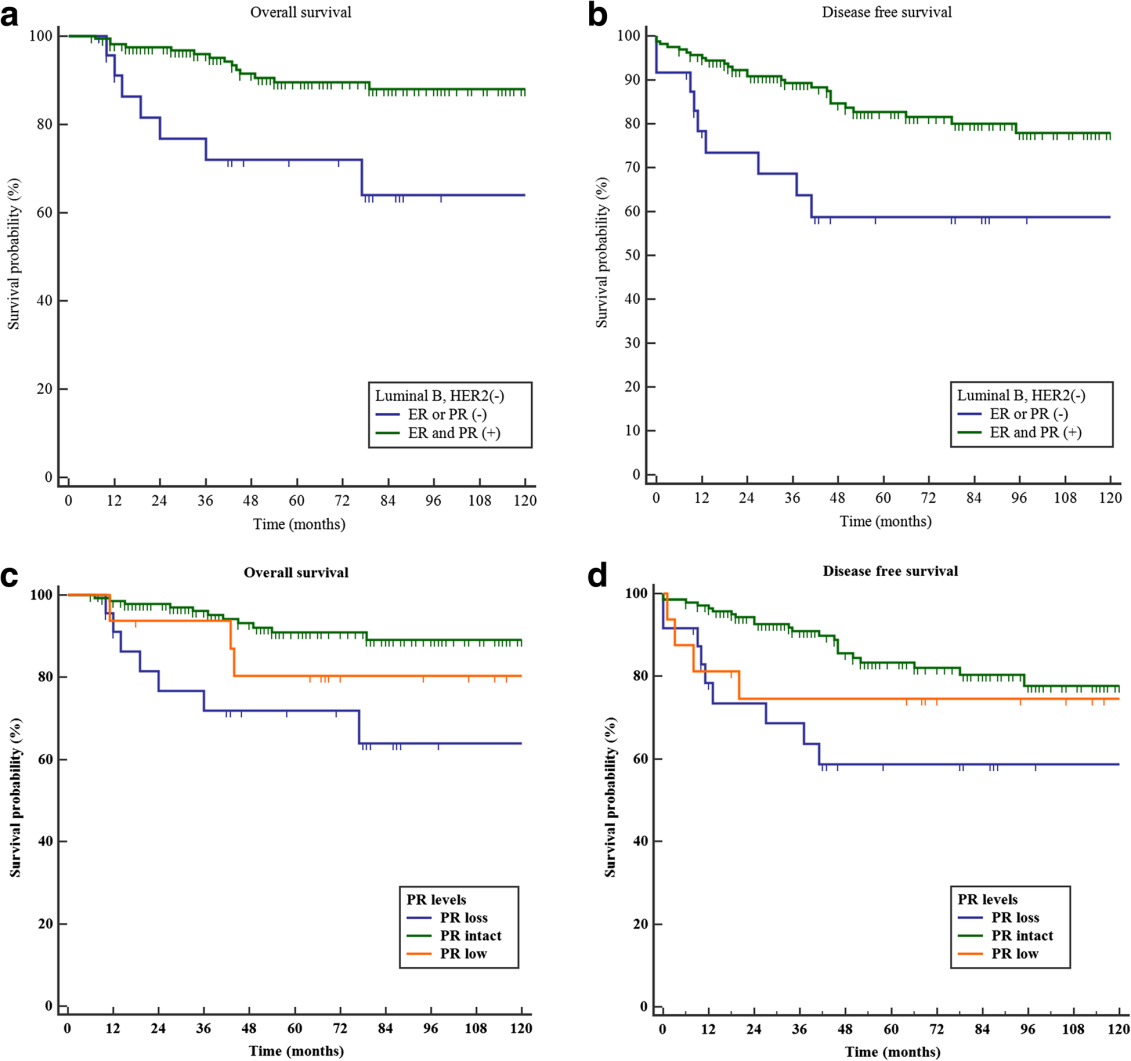
**a** Overall survivals of ER- or PR-negative group and ER- and PR-positive group in the luminal B HER-2 negative breast cancer (*p* = 0.0119). **b** Disease-free survivals of ER- or PR-negative group and ER- and PR-positive group in the luminal B HER-2-negative breast cancer (*p* = 0.0338). **c** Overall survivals of PR-positive, PR low, and PR-negative group in the luminal B HER-2-negative breast cancer (*p* < 0.0001). **d** Disease-free survivals of PR-positive, PR low, and PR-negative group in the luminal B HER-2-negative breast cancer (*p* = 0.0005) (Additional file 1)

To find out the impact of ER or PR negativity to survival of the luminal B HER2-negative breast cancer, we analyzed DFS and OS according to the ER or PR status. We could see the facts that DFS and OS were better in the ER- and PR-positive group than those in the ER- or PR-negative group, and there were statistically significant differences between the two groups (*p* = 0.0338, *p* = 0.0119, respectively) (Fig. 1a, b).

### DFS and OS according to negative, low expression, or positive PR status

We were also interested in whether PR expression level was associated with prognosis. Thus, we divided the PR-positive group into low and high PR expression groups and performed a survival analysis according to PR expression level. The results showed that low PR expression group resulted in better DFS (*p* = 0.0005) and OS (*p* < 0.0001) than those of the negative PR expression group but was worse than that of the high PR expression group (Fig. 1c, d).

### DFS and OS according to Bcl-2 expression status

**Fig. 2:**
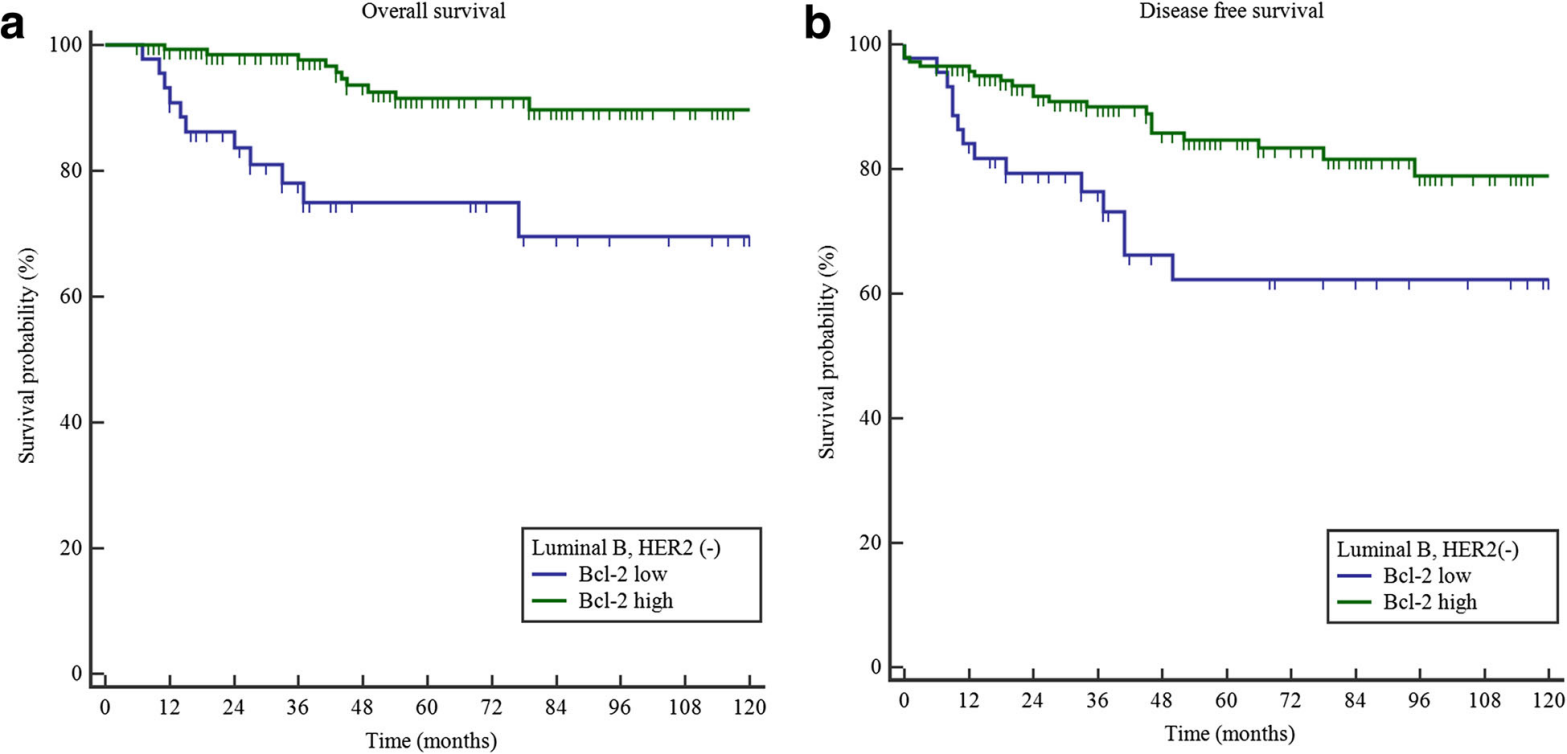
**a** Overall survivals of low and high Bcl-2 expression group in the luminal B HER-2-negative breast cancer (*p* < 0.0001). **b** Disease-free survivals of low and high Bcl-2 expression group in the luminal B HER-2-negative breast cancer (*p* = 0.0007) (Additional file 1)

As we mentioned above, negative ER or PR expression group showed shorter DFS and OS than positive ER and PR expression group. In our multivariate analysis, Bcl-2 expression also was a prognostic factor related to ER or PR expression status. When we analyzed DFS and OS according to the expression status of Bcl-2, lower Bcl-2 expression has relation with better DFS (*p* = 0.0007) and OS (*p* < 0.0001) (Fig. 2).

## Discussion

The ER and PR are expressed in approximately 60~70 % of breast carcinomas, suggesting that steroid hormones influence tumor progression [13]. Systematic investigations into the gene expression patterns in human breast tumors have provided the basis for improved molecular taxonomy of breast cancers [21]. Among the four alleged molecular subtypes of breast cancer, the luminal subtype expresses ER and/or PR, which is a prognostic factor and predictive marker for anti-estrogen hormonal therapies, such as SERM or aromatase inhibitors. However, the luminal subtype of breast cancer is very heterogeneous, and its certain subsets have been showing very aggressive characteristics in clinical setting. Sometimes, their prognosis is similar to or worse than that of HER-2-enriched or triple-negative breast cancers [22]. Bardou et al. had worked with breast cancer databases to investigate whether PR status provides additional value to ER status in patients with primary breast cancer. The results indicated that PR status is an independent predictive factor for a benefit from adjuvant anti-estrogen therapy [23].

Our investigators wanted to know the clinical implications of the negativity of ER or PR in luminal HER2-negative breast cancer from this study. Our results showed that ER or PR negativity is poor prognostic factors, and disease-free survival and overall survival in the ER- or PR-negative group were shorter than that in the ER- and PR-positive group. We also wanted to know the specific role of each hormonal receptor through the comparison between the ER-negative and PR-negative groups. As a result, the ER-negative group tended to have shorter survival than the PR-negative group. Few studies were conducted on ER-negative and PR-positive breast cancer because of the low incidence of cases. These studies showed that ER-negative and PR-positive breast cancer is associated with older age, higher proliferation, and a worse prognosis [22, 24]. ER-positive and PR-negative tumors differ from ER-negative and PR-positive tumors, as shown by our results. When we analyzed the prognostic value according to the level of PR expression, the low PR expression group showed an intermediate prognosis better than the negative PR and worse than the high PR expression group. Nishimukai et al. reported that low PR expression is associated with prognosis of ER-positive and HER2-negative breast cancer [25], which agrees with our study.

Furthermore, the Arimidex, Tamoxifen, Alone or in Combination (ATAC) trial showed that patients with ER-positive and PR-negative tumors have a higher recurrence rate than those with ER-positive and PR-positive tumors. In a subgroup analysis of ER-positive and PR-negative tumors, the recurrence rate was much higher in the group that received tamoxifen than that in the group that received anastrozole [26, 27]. Growing evidence supports that ER-positive and PR-negative breast cancers are less responsive to SERM than that of ER-positive and PR-positive tumors. Because increased crosstalk between ER and growth factor signaling pathways leads to downregulated PR transcription, blocking the ER completely with aromatase inhibitor could be more effective in patients with ER-positive and PR-negative breast cancer [28]. In our study, we have specified a luminal B HER2-negative breast cancer subgroup to determine whether ER or PR negativity provides additional prognostic value in patients with high risk HER2-negative endocrine-responsive breast cancer. As a result, the ER- or PR-negative group showed a higher recurrence rate and decreased OS compared to the ER- and PR-positive group. However, we found no difference when we compared anastrozole to tamoxifen in our study because of the small number of patients.

Several years ago, we had reported the results of study that Bcl-2 may be a potent prognostic factor in patients with luminal subtype of breast cancer [17]. Sivestrini et al. had reported that expression of Bcl-2, an anti-apoptotic protein, is associated with low-grade, slowly proliferating, ER-positive breast tumors [29]. There are several previous studies had shown that increased expression of Bcl-2 was associated with improved survival in breast cancer and probability of prognostic role in endocrine-responsive breast cancer [30, 31]. In our current study, we also could see the common results that lower Bcl-2 expression is related with shorter survival and ER or PR negativity, suggesting that Bcl-2 may be a candidate prognostic factor in patients with endocrine-responsive breast cancer. There is a study that when patients were classified into four groups based on HR and HER2 status, more than 10 % of Bcl-2 expression in the HR-positive HER2-negative group resulted in a poorer prognosis, which agrees with our results [32]. We authors cautiously suggest that Bcl-2 expression might have a prognostic role in luminal B HER2-negative breast cancer from our current study.

Through the recent studies for gene expression profile in breast cancer, we had an insight for the role of proliferative signatures in breast cancer in terms of prognosis and prediction of response to anti-cancer therapy [33]. Ki-67 expression levels varies throughout the different cell cycle phases, which are low in the G1 and S phases and reaches its peak level in mitosis [34]. The panel of the St Gallen International Expert Consensus on the primary therapy of early breast cancer recommends the use of proliferation markers such as Ki-67 index when physicians decide the appropriate systemic treatment in addition to traditional parameters [35]. However, there are some arguments between investigators to determine the appropriate cutoff values of Ki-67 in terms of prognosis of breast cancer or finding out the most potent subgroup who may get more advantages from anti-cancer therapy.

Although the role of Ki-67 as a prognostic factor is controversial in breast cancer, many studies have shown a relationship between Ki-67 and HR [25, 36]. When we divided luminal B HER2-negative breast cancer patients into two subgroups according to a Ki-67 level with cutoff 50 %, the subgroup with higher Ki-67 (>50 %) was related with the negative ER or PR group. Nishimukai et al. reported that high Ki-67 expression and low PR expression is associated with the prognosis of patients with ER-positive HER2-negative cancers [25], which agrees with our results.

## Conclusions

ER or PR negativities in patients with luminal B HER2-negative breast cancer were strongly associated with a poor prognosis. We observed that ER or PR negativities are more frequent in patients with luminal B HER2-negative breast cancer who were older than 45 years old, had lower Bcl-2 expression, and a higher Ki 67 index. However, additional studies with large number of patients who are classified to luminal B HER2-negative breast cancer subgroup might reveal the role of ER or PR negativities in breast cancer.

## Additional file

Additional file 1: Result of statistic analysis. (DOCX 389 kb)

## Abbreviations

HER2: Human epidermal growth factor receptor2
ER: Estrogen receptor
PR: Progesterone receptor
DFS: Disease-free survival
OS: Overall survival
EGFR: Epidermal growth factor receptor
IHC: Immunohistochemical
SISH: Silver-enhanced in situ hybridization
SERM: Selective estrogen receptor modulator
