# Supplementary material for: Prognostic values of negative estrogen or progesterone receptor expression in patients with luminal B HER2-negative breast cancer
Source: World J Surg Oncol. 2016 Sep 13;14(1):244. doi: 10.1186/s12957-016-0999-x (PMC5020449; doi:10.1186/s12957-016-0999-x)

# Kaplan-Meier survival analysis

| Survival time | os |
| --- | --- |
| Endpoint | 사망유무__death__1__live__0 |
| Factor codes | ERPR_loss |

## Cases summary

|  | Number of events ^a^ | | Number censored ^b^ | |  |
| --- | --- | --- | --- | --- | --- |
| Factor | N | % | N | % | Total sample size |
| ERPR loss | 7 | 29.17 | 17 | 70.83 | 24 |
| LumB | 14 | 8.75 | 146 | 91.25 | 160 |
| Overall | 21 | 11.41 | 163 | 88.59 | 184 |

^a^ 사망유무__death__1__live__0 = 1
^b^ 사망유무__death__1__live__0 = 0

## Mean and median survival

| Factor | Mean | SE | 95% CI for the mean | Median | 95% CI for the median |
| --- | --- | --- | --- | --- | --- |
| ERPR loss | 88.946 | 10.032 | 69.283 to 108.610 | - | - |
| LumB | 124.546 | 2.898 | 118.866 to 130.226 | - | - |
| Overall | 121.124 | 3.026 | 115.192 to 127.055 | - | - |

## Survival table [[Show]](javascript:showdiv('d16','d17','table1');)

## Survival table [[Hide]](javascript:hidediv('d16','d17','table1');)

|  | Factor | | | |  | |
| --- | --- | --- | --- | --- | --- | --- |
|  | ERPR loss | | LumB | | Overall | |
| Survival time | Survival Proportion | Standard Error | Survival Proportion | Standard Error | Survival Proportion | Standard Error |
| 6 | - | - | - | - | - | - |
| 7 | - | - | 0.994 | 0.00627 | 0.995 | 0.00545 |
| 8 | - | - | - | - | - | - |
| 9 | - | - | - | - | - | - |
| 10 | 0.957 | 0.0425 | - | - | 0.989 | 0.00773 |
| 11 | - | - | 0.981 | 0.0108 | 0.978 | 0.0109 |
| 12 | 0.911 | 0.0601 | - | - | 0.972 | 0.0122 |
| 14 | 0.863 | 0.0736 | - | - | 0.967 | 0.0134 |
| 15 | - | - | 0.975 | 0.0125 | 0.961 | 0.0145 |
| 16 | - | - | - | - | - | - |
| 17 | - | - | - | - | - | - |
| 18 | - | - | - | - | - | - |
| 19 | 0.815 | 0.0837 | - | - | 0.955 | 0.0155 |
| 20 | - | - | - | - | - | - |
| 21 | - | - | - | - | - | - |
| 22 | - | - | - | - | - | - |
| 24 | 0.767 | 0.0915 | - | - | 0.949 | 0.0166 |
| 25 | - | - | - | - | - | - |
| 26 | - | - | - | - | - | - |
| 27 | - | - | 0.967 | 0.0145 | 0.942 | 0.0177 |
| 28 | - | - | - | - | - | - |
| 29 | - | - | - | - | - | - |
| 30 | - | - | - | - | - | - |
| 31 | - | - | - | - | - | - |
| 32 | - | - | - | - | - | - |
| 33 | - | - | 0.959 | 0.0163 | 0.936 | 0.0188 |
| 34 | - | - | - | - | - | - |
| 36 | 0.719 | 0.0975 | - | - | 0.929 | 0.0199 |
| 37 | - | - | 0.951 | 0.0182 | 0.922 | 0.0210 |
| 38 | - | - | - | - | - | - |
| 39 | - | - | - | - | - | - |
| 40 | - | - | - | - | - | - |
| 41 | - | - | 0.942 | 0.0200 | 0.914 | 0.0221 |
| 42 | - | - | - | - | - | - |
| 43 | - | - | 0.933 | 0.0217 | 0.907 | 0.0232 |
| 44 | - | - | 0.924 | 0.0233 | 0.899 | 0.0243 |
| 45 | - | - | 0.915 | 0.0248 | 0.891 | 0.0253 |
| 46 | - | - | - | - | - | - |
| 48 | - | - | - | - | - | - |
| 49 | - | - | 0.906 | 0.0262 | 0.883 | 0.0264 |
| 50 | - | - | - | - | - | - |
| 51 | - | - | - | - | - | - |
| 52 | - | - | - | - | - | - |
| 53 | - | - | - | - | - | - |
| 54 | - | - | 0.896 | 0.0277 | 0.874 | 0.0275 |
| 55 | - | - | - | - | - | - |
| 56 | - | - | - | - | - | - |
| 57 | - | - | - | - | - | - |
| 58 | - | - | - | - | - | - |
| 59 | - | - | - | - | - | - |
| 61 | - | - | - | - | - | - |
| 62 | - | - | - | - | - | - |
| 63 | - | - | - | - | - | - |
| 64 | - | - | - | - | - | - |
| 66 | - | - | - | - | - | - |
| 67 | - | - | - | - | - | - |
| 68 | - | - | - | - | - | - |
| 69 | - | - | - | - | - | - |
| 71 | - | - | - | - | - | - |
| 72 | - | - | - | - | - | - |
| 74 | - | - | - | - | - | - |
| 76 | - | - | - | - | - | - |
| 77 | 0.639 | 0.115 | - | - | 0.861 | 0.0301 |
| 78 | - | - | - | - | - | - |
| 79 | - | - | 0.880 | 0.0315 | 0.847 | 0.0326 |
| 80 | - | - | - | - | - | - |
| 81 | - | - | - | - | - | - |
| 83 | - | - | - | - | - | - |
| 84 | - | - | - | - | - | - |
| 85 | - | - | - | - | - | - |
| 86 | - | - | - | - | - | - |
| 87 | - | - | - | - | - | - |
| 88 | - | - | - | - | - | - |
| 89 | - | - | - | - | - | - |
| 91 | - | - | - | - | - | - |
| 92 | - | - | - | - | - | - |
| 94 | - | - | - | - | - | - |
| 96 | - | - | - | - | - | - |
| 97 | - | - | - | - | - | - |
| 98 | - | - | - | - | - | - |
| 99 | - | - | - | - | - | - |
| 100 | - | - | - | - | - | - |
| 102 | - | - | - | - | - | - |
| 105 | - | - | - | - | - | - |
| 106 | - | - | - | - | - | - |
| 109 | - | - | - | - | - | - |
| 110 | - | - | - | - | - | - |
| 113 | - | - | - | - | - | - |
| 114 | - | - | - | - | - | - |
| 115 | - | - | - | - | - | - |
| 116 | - | - | - | - | - | - |
| 117 | - | - | - | - | - | - |
| 119 | - | - | - | - | - | - |
| 120 | - | - | - | - | - | - |
| 121 | - | - | - | - | - | - |
| 122 | - | - | - | - | - | - |
| 125 | - | - | - | - | - | - |
| 127 | - | - | - | - | - | - |
| 129 | - | - | - | - | - | - |
| 130 | - | - | - | - | - | - |
| 132 | - | - | - | - | - | - |
| 136 | - | - | - | - | - | - |
| Endpoint: Observed n | 7.0 | | 14.0 | |  | |
| Expected n | 2.4 | | 18.6 | |  | |
| Observed/Expected | 2.9021 | | 0.7532 | |  | |

## Comparison of survival curves (Logrank test)

| Chi-squared | 9.8721 |
| --- | --- |
| DF | 1 |
| Significance | P = 0.0017 |

## Hazard ratios^a^ with 95% Confidence Interval

| Factor | ERPR loss | LumB |
| --- | --- | --- |
| ERPR loss | - | 0.2595 0.06786 to 0.9925 |
| LumB | 3.8531 1.0075 to 14.7358 | - |

^a^ Column/Row


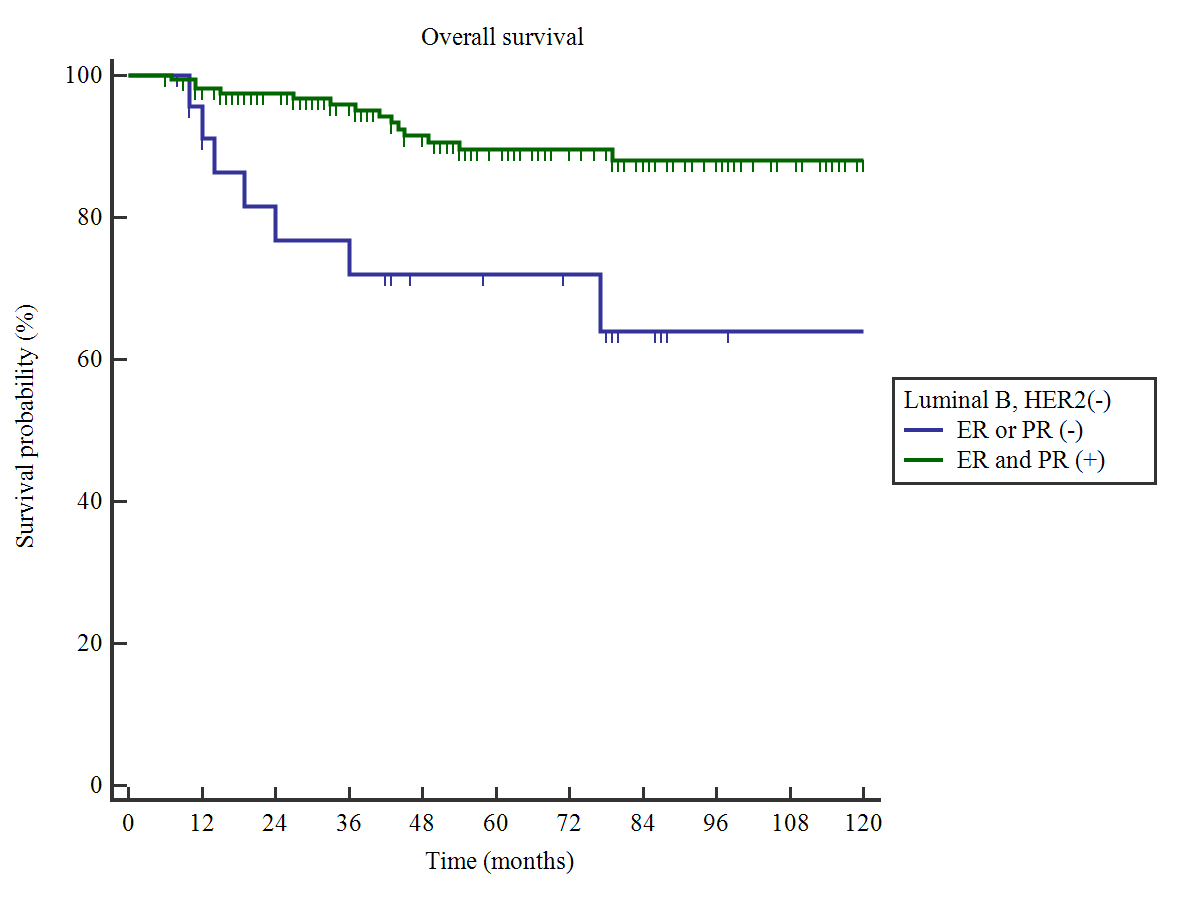


# Kaplan-Meier survival analysis

| Survival time | dfs |
| --- | --- |
| Endpoint | 재발_유무 |
| Factor codes | ERPR_loss |

## Cases summary

|  | Number of events ^a^ | | Number censored ^b^ | |  |
| --- | --- | --- | --- | --- | --- |
| Factor | N | % | N | % | Total sample size |
| ERPR loss | 9 | 37.50 | 15 | 62.50 | 24 |
| LumB | 26 | 16.25 | 134 | 83.75 | 160 |
| Overall | 35 | 19.02 | 149 | 80.98 | 184 |

^a^ 재발_유무 = 1
^b^ 재발_유무 = 0

## Mean and median survival

| Factor | Mean | SE | 95% CI for the mean | Median | 95% CI for the median |
| --- | --- | --- | --- | --- | --- |
| ERPR loss | 78.196 | 11.131 | 56.380 to 100.013 | - | - |
| LumB | 114.561 | 3.797 | 107.120 to 122.002 | - | - |
| Overall | 110.865 | 3.775 | 103.466 to 118.263 | - | - |

## Survival table [[Show]](javascript:showdiv('d18','d19','table1');)

## Survival table [[Hide]](javascript:hidediv('d18','d19','table1');)

|  | Factor | | | |  | |
| --- | --- | --- | --- | --- | --- | --- |
|  | ERPR loss | | LumB | | Overall | |
| Survival time | Survival Proportion | Standard Error | Survival Proportion | Standard Error | Survival Proportion | Standard Error |
| 0 | 0.917 | 0.0564 | 0.988 | 0.00878 | 0.978 | 0.0108 |
| 1 | - | - | 0.981 | 0.0107 | 0.973 | 0.0120 |
| 3 | - | - | 0.975 | 0.0123 | 0.967 | 0.0131 |
| 6 | - | - | 0.969 | 0.0138 | 0.962 | 0.0141 |
| 8 | - | - | 0.962 | 0.0150 | 0.956 | 0.0150 |
| 9 | 0.873 | 0.0686 | 0.956 | 0.0162 | 0.945 | 0.0168 |
| 10 | 0.829 | 0.0778 | - | - | 0.940 | 0.0176 |
| 11 | 0.783 | 0.0860 | - | - | 0.934 | 0.0183 |
| 12 | - | - | 0.950 | 0.0173 | 0.929 | 0.0190 |
| 13 | 0.734 | 0.0936 | 0.943 | 0.0183 | 0.917 | 0.0204 |
| 14 | - | - | - | - | - | - |
| 15 | - | - | - | - | - | - |
| 16 | - | - | - | - | - | - |
| 17 | - | - | - | - | - | - |
| 18 | - | - | 0.937 | 0.0194 | 0.912 | 0.0211 |
| 19 | - | - | 0.930 | 0.0204 | 0.906 | 0.0218 |
| 20 | - | - | 0.923 | 0.0214 | 0.900 | 0.0225 |
| 21 | - | - | - | - | - | - |
| 22 | - | - | - | - | - | - |
| 24 | - | - | 0.909 | 0.0234 | 0.887 | 0.0239 |
| 25 | - | - | - | - | - | - |
| 26 | - | - | - | - | - | - |
| 27 | 0.685 | 0.0993 | - | - | 0.881 | 0.0246 |
| 28 | - | - | - | - | - | - |
| 29 | - | - | - | - | - | - |
| 30 | - | - | - | - | - | - |
| 31 | - | - | - | - | - | - |
| 32 | - | - | - | - | - | - |
| 33 | - | - | 0.901 | 0.0244 | 0.874 | 0.0253 |
| 34 | - | - | 0.893 | 0.0255 | 0.867 | 0.0260 |
| 36 | - | - | - | - | - | - |
| 37 | 0.636 | 0.104 | - | - | 0.860 | 0.0268 |
| 38 | - | - | - | - | - | - |
| 39 | - | - | - | - | - | - |
| 40 | - | - | - | - | - | - |
| 41 | 0.587 | 0.107 | 0.884 | 0.0268 | 0.844 | 0.0284 |
| 42 | - | - | - | - | - | - |
| 43 | - | - | - | - | - | - |
| 45 | - | - | 0.874 | 0.0281 | 0.836 | 0.0293 |
| 46 | - | - | 0.847 | 0.0315 | 0.812 | 0.0316 |
| 48 | - | - | - | - | - | - |
| 50 | - | - | 0.837 | 0.0325 | 0.803 | 0.0324 |
| 52 | - | - | 0.827 | 0.0335 | 0.795 | 0.0331 |
| 53 | - | - | - | - | - | - |
| 54 | - | - | - | - | - | - |
| 55 | - | - | - | - | - | - |
| 56 | - | - | - | - | - | - |
| 57 | - | - | - | - | - | - |
| 58 | - | - | - | - | - | - |
| 59 | - | - | - | - | - | - |
| 62 | - | - | - | - | - | - |
| 63 | - | - | - | - | - | - |
| 64 | - | - | - | - | - | - |
| 66 | - | - | 0.816 | 0.0350 | 0.785 | 0.0342 |
| 67 | - | - | - | - | - | - |
| 68 | - | - | - | - | - | - |
| 69 | - | - | - | - | - | - |
| 72 | - | - | - | - | - | - |
| 74 | - | - | - | - | - | - |
| 76 | - | - | - | - | - | - |
| 78 | - | - | 0.801 | 0.0375 | 0.772 | 0.0360 |
| 79 | - | - | - | - | - | - |
| 80 | - | - | - | - | - | - |
| 81 | - | - | - | - | - | - |
| 83 | - | - | - | - | - | - |
| 84 | - | - | - | - | - | - |
| 85 | - | - | - | - | - | - |
| 86 | - | - | - | - | - | - |
| 87 | - | - | - | - | - | - |
| 88 | - | - | - | - | - | - |
| 89 | - | - | - | - | - | - |
| 91 | - | - | - | - | - | - |
| 92 | - | - | - | - | - | - |
| 94 | - | - | - | - | - | - |
| 95 | - | - | 0.778 | 0.0425 | 0.752 | 0.0404 |
| 96 | - | - | - | - | - | - |
| 97 | - | - | - | - | - | - |
| 98 | - | - | - | - | - | - |
| 99 | - | - | - | - | - | - |
| 100 | - | - | - | - | - | - |
| 102 | - | - | - | - | - | - |
| 105 | - | - | - | - | - | - |
| 106 | - | - | - | - | - | - |
| 109 | - | - | - | - | - | - |
| 110 | - | - | - | - | - | - |
| 113 | - | - | - | - | - | - |
| 114 | - | - | - | - | - | - |
| 115 | - | - | - | - | - | - |
| 116 | - | - | - | - | - | - |
| 117 | - | - | - | - | - | - |
| 119 | - | - | - | - | - | - |
| 120 | - | - | - | - | - | - |
| 121 | - | - | - | - | - | - |
| 125 | - | - | - | - | - | - |
| 127 | - | - | - | - | - | - |
| 129 | - | - | - | - | - | - |
| 130 | - | - | - | - | - | - |
| 132 | - | - | - | - | - | - |
| 136 | - | - | - | - | - | - |
| Endpoint: Observed n | 9.0 | | 26.0 | |  | |
| Expected n | 3.7 | | 31.3 | |  | |
| Observed/Expected | 2.4118 | | 0.8315 | |  | |

## Comparison of survival curves (Logrank test)

| Chi-squared | 8.3966 |
| --- | --- |
| DF | 1 |
| Significance | P = 0.0038 |

## Hazard ratios^a^ with 95% Confidence Interval

| Factor | ERPR loss | LumB |
| --- | --- | --- |
| ERPR loss | - | 0.3448 0.1178 to 1.0086 |
| LumB | 2.9005 0.9915 to 8.4855 | - |

^a^ Column/Row


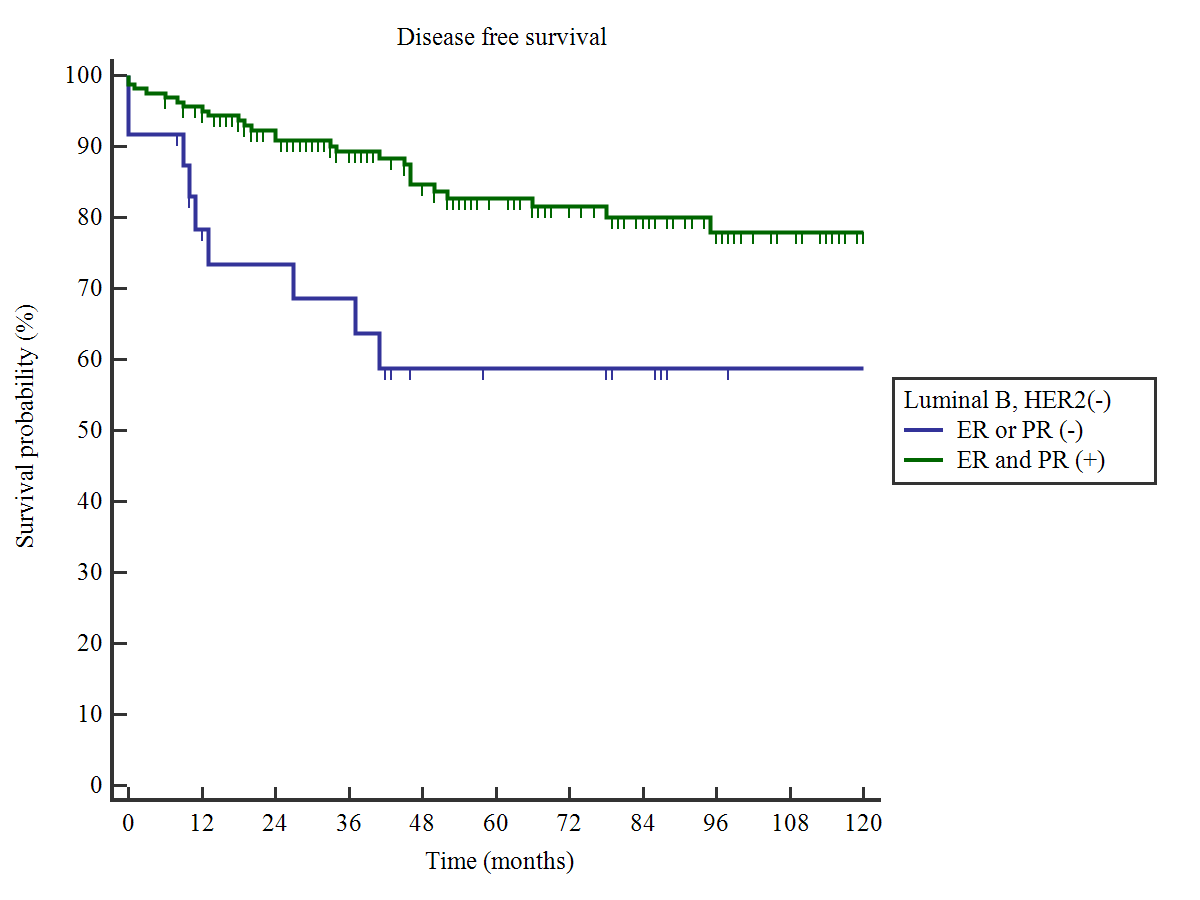


# Kaplan-Meier survival analysis

| Survival time | os |
| --- | --- |
| Endpoint | 사망유무__death__1__live__0 |
| Factor codes | ERPR_low_5% |

## Cases summary

|  | Number of events ^a^ | | Number censored ^b^ | |  |
| --- | --- | --- | --- | --- | --- |
| Factor | N | % | N | % | Total sample size |
| loss | 7 | 29.17 | 17 | 70.83 | 24 |
| LumB | 11 | 7.64 | 133 | 92.36 | 144 |
| PR low | 3 | 18.75 | 13 | 81.25 | 16 |
| Overall | 21 | 11.41 | 163 | 88.59 | 184 |

^a^ 사망유무__death__1__live__0 = 1
^b^ 사망유무__death__1__live__0 = 0

## Mean and median survival

| Factor | Mean | SE | 95% CI for the mean | Median | 95% CI for the median |
| --- | --- | --- | --- | --- | --- |
| loss | 88.946 | 10.032 | 69.283 to 108.610 | - | - |
| LumB | 125.756 | 2.944 | 119.986 to 131.525 | - | - |
| PR low | 110.978 | 9.958 | 91.459 to 130.496 | - | - |
| Overall | 121.124 | 3.026 | 115.192 to 127.055 | - | - |

## Survival table [[Show]](javascript:showdiv('d0','d1','table1');)

## Survival table [[Hide]](javascript:hidediv('d0','d1','table1');)

|  | Factor | | | | | |  | |
| --- | --- | --- | --- | --- | --- | --- | --- | --- |
|  | loss | | LumB | | PR low | | Overall | |
| Survival time | Survival Proportion | Standard Error | Survival Proportion | Standard Error | Survival Proportion | Standard Error | Survival Proportion | Standard Error |
| 6 | - | - | - | - | - | - | - | - |
| 7 | - | - | 0.993 | 0.00697 | - | - | 0.995 | 0.00545 |
| 8 | - | - | - | - | - | - | - | - |
| 9 | - | - | - | - | - | - | - | - |
| 10 | 0.957 | 0.0425 | - | - | - | - | 0.989 | 0.00773 |
| 11 | - | - | 0.986 | 0.00985 | 0.938 | 0.0605 | 0.978 | 0.0109 |
| 12 | 0.911 | 0.0601 | - | - | - | - | 0.972 | 0.0122 |
| 14 | 0.863 | 0.0736 | - | - | - | - | 0.967 | 0.0134 |
| 15 | - | - | 0.979 | 0.0121 | - | - | 0.961 | 0.0145 |
| 16 | - | - | - | - | - | - | - | - |
| 17 | - | - | - | - | - | - | - | - |
| 18 | - | - | - | - | - | - | - | - |
| 19 | 0.815 | 0.0837 | - | - | - | - | 0.955 | 0.0155 |
| 20 | - | - | - | - | - | - | - | - |
| 21 | - | - | - | - | - | - | - | - |
| 22 | - | - | - | - | - | - | - | - |
| 24 | 0.767 | 0.0915 | - | - | - | - | 0.949 | 0.0166 |
| 25 | - | - | - | - | - | - | - | - |
| 26 | - | - | - | - | - | - | - | - |
| 27 | - | - | 0.970 | 0.0146 | - | - | 0.942 | 0.0177 |
| 28 | - | - | - | - | - | - | - | - |
| 29 | - | - | - | - | - | - | - | - |
| 30 | - | - | - | - | - | - | - | - |
| 31 | - | - | - | - | - | - | - | - |
| 32 | - | - | - | - | - | - | - | - |
| 33 | - | - | 0.962 | 0.0169 | - | - | 0.936 | 0.0188 |
| 34 | - | - | - | - | - | - | - | - |
| 36 | 0.719 | 0.0975 | - | - | - | - | 0.929 | 0.0199 |
| 37 | - | - | 0.952 | 0.0192 | - | - | 0.922 | 0.0210 |
| 38 | - | - | - | - | - | - | - | - |
| 39 | - | - | - | - | - | - | - | - |
| 40 | - | - | - | - | - | - | - | - |
| 41 | - | - | 0.942 | 0.0215 | - | - | 0.914 | 0.0221 |
| 42 | - | - | - | - | - | - | - | - |
| 43 | - | - | - | - | 0.871 | 0.0856 | 0.907 | 0.0232 |
| 44 | - | - | - | - | 0.804 | 0.102 | 0.899 | 0.0243 |
| 45 | - | - | 0.932 | 0.0237 | - | - | 0.891 | 0.0253 |
| 46 | - | - | - | - | - | - | - | - |
| 48 | - | - | - | - | - | - | - | - |
| 49 | - | - | 0.921 | 0.0258 | - | - | 0.883 | 0.0264 |
| 50 | - | - | - | - | - | - | - | - |
| 51 | - | - | - | - | - | - | - | - |
| 52 | - | - | - | - | - | - | - | - |
| 53 | - | - | - | - | - | - | - | - |
| 54 | - | - | 0.909 | 0.0279 | - | - | 0.874 | 0.0275 |
| 55 | - | - | - | - | - | - | - | - |
| 56 | - | - | - | - | - | - | - | - |
| 57 | - | - | - | - | - | - | - | - |
| 58 | - | - | - | - | - | - | - | - |
| 59 | - | - | - | - | - | - | - | - |
| 61 | - | - | - | - | - | - | - | - |
| 62 | - | - | - | - | - | - | - | - |
| 63 | - | - | - | - | - | - | - | - |
| 64 | - | - | - | - | - | - | - | - |
| 66 | - | - | - | - | - | - | - | - |
| 67 | - | - | - | - | - | - | - | - |
| 68 | - | - | - | - | - | - | - | - |
| 69 | - | - | - | - | - | - | - | - |
| 71 | - | - | - | - | - | - | - | - |
| 72 | - | - | - | - | - | - | - | - |
| 74 | - | - | - | - | - | - | - | - |
| 76 | - | - | - | - | - | - | - | - |
| 77 | 0.639 | 0.115 | - | - | - | - | 0.861 | 0.0301 |
| 78 | - | - | - | - | - | - | - | - |
| 79 | - | - | 0.891 | 0.0327 | - | - | 0.847 | 0.0326 |
| 80 | - | - | - | - | - | - | - | - |
| 81 | - | - | - | - | - | - | - | - |
| 83 | - | - | - | - | - | - | - | - |
| 84 | - | - | - | - | - | - | - | - |
| 85 | - | - | - | - | - | - | - | - |
| 86 | - | - | - | - | - | - | - | - |
| 87 | - | - | - | - | - | - | - | - |
| 88 | - | - | - | - | - | - | - | - |
| 89 | - | - | - | - | - | - | - | - |
| 91 | - | - | - | - | - | - | - | - |
| 92 | - | - | - | - | - | - | - | - |
| 94 | - | - | - | - | - | - | - | - |
| 96 | - | - | - | - | - | - | - | - |
| 97 | - | - | - | - | - | - | - | - |
| 98 | - | - | - | - | - | - | - | - |
| 99 | - | - | - | - | - | - | - | - |
| 100 | - | - | - | - | - | - | - | - |
| 102 | - | - | - | - | - | - | - | - |
| 105 | - | - | - | - | - | - | - | - |
| 106 | - | - | - | - | - | - | - | - |
| 109 | - | - | - | - | - | - | - | - |
| 110 | - | - | - | - | - | - | - | - |
| 113 | - | - | - | - | - | - | - | - |
| 114 | - | - | - | - | - | - | - | - |
| 115 | - | - | - | - | - | - | - | - |
| 116 | - | - | - | - | - | - | - | - |
| 117 | - | - | - | - | - | - | - | - |
| 119 | - | - | - | - | - | - | - | - |
| 120 | - | - | - | - | - | - | - | - |
| 121 | - | - | - | - | - | - | - | - |
| 122 | - | - | - | - | - | - | - | - |
| 125 | - | - | - | - | - | - | - | - |
| 127 | - | - | - | - | - | - | - | - |
| 129 | - | - | - | - | - | - | - | - |
| 130 | - | - | - | - | - | - | - | - |
| 132 | - | - | - | - | - | - | - | - |
| 136 | - | - | - | - | - | - | - | - |
| Endpoint: Observed n | 7.0 | | 11.0 | | 3.0 | |  | |
| Expected n | 2.4 | | 16.5 | | 2.1 | |  | |
| Observed/Expected | 2.9021 | | 0.6656 | | 1.4552 | |  | |

## Comparison of survival curves (Logrank test)

| Chi-squared | 11.0151 |
| --- | --- |
| DF | 2 |
| Significance | P = 0.0041 |

## Hazard ratios^a^ with 95% Confidence Interval

| Factor | loss | LumB | PR low |
| --- | --- | --- | --- |
| loss | - | 0.2294 0.05940 to 0.8856 | 0.5014 0.07813 to 3.2181 |
| LumB | 4.3601 1.1292 to 16.8351 | - | 2.1863 0.5140 to 9.2992 |
| PR low | 1.9943 0.3107 to 12.7987 | 0.4574 0.1075 to 1.9455 | - |

^a^ Column/Row


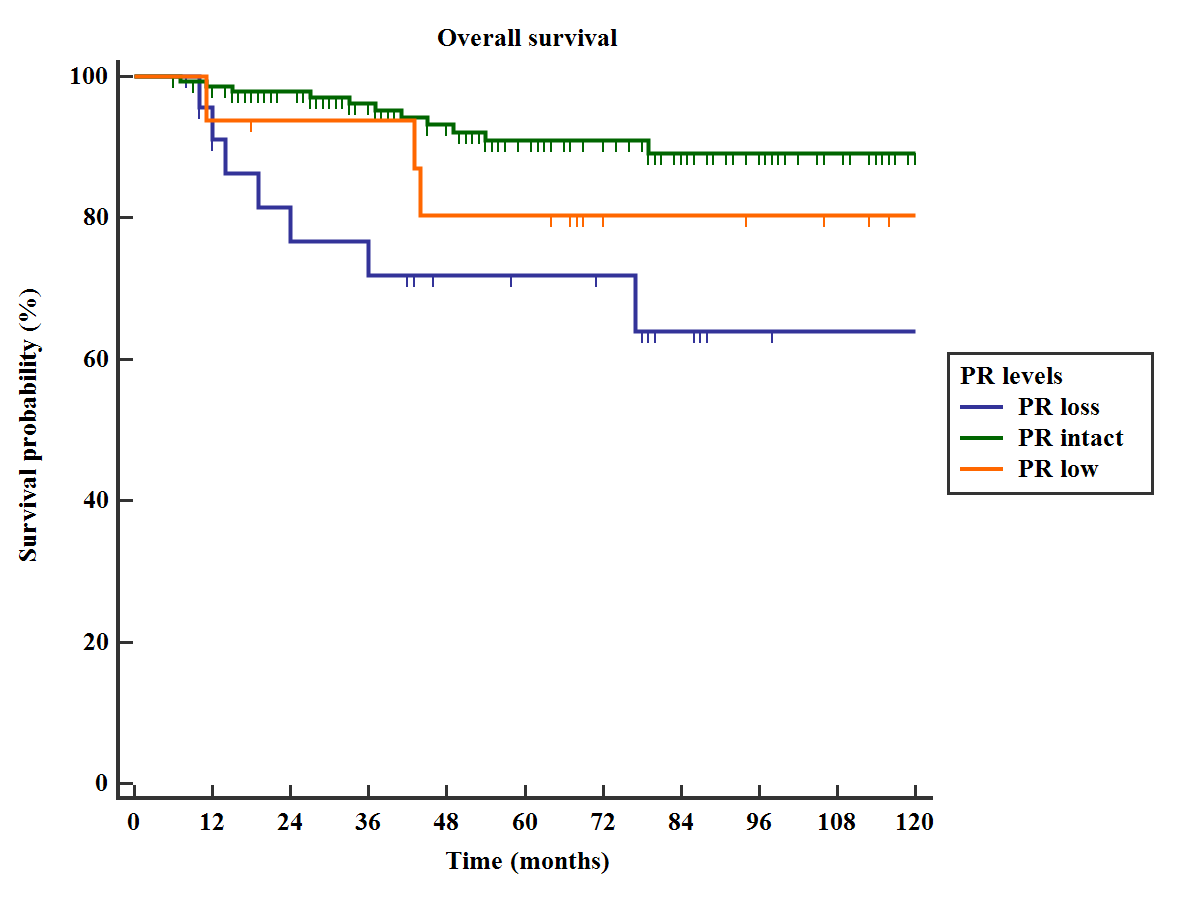


# Kaplan-Meier survival analysis

| Survival time | dfs |
| --- | --- |
| Endpoint | 재발_유무 |
| Factor codes | ERPR_low_5% |

## Cases summary

|  | Number of events ^a^ | | Number censored ^b^ | |  |
| --- | --- | --- | --- | --- | --- |
| Factor | N | % | N | % | Total sample size |
| loss | 9 | 37.50 | 15 | 62.50 | 24 |
| LumB | 22 | 15.28 | 122 | 84.72 | 144 |
| PR low | 4 | 25.00 | 12 | 75.00 | 16 |
| Overall | 35 | 19.02 | 149 | 80.98 | 184 |

^a^ 재발_유무 = 1
^b^ 재발_유무 = 0

## Mean and median survival

| Factor | Mean | SE | 95% CI for the mean | Median | 95% CI for the median |
| --- | --- | --- | --- | --- | --- |
| loss | 78.196 | 11.131 | 56.380 to 100.013 | - | - |
| LumB | 115.427 | 3.965 | 107.656 to 123.198 | - | - |
| PR low | 98.927 | 13.451 | 72.564 to 125.290 | - | - |
| Overall | 110.865 | 3.775 | 103.466 to 118.263 | - | - |

## Survival table [[Show]](javascript:showdiv('d2','d3','table1');)

## Survival table [[Hide]](javascript:hidediv('d2','d3','table1');)

|  | Factor | | | | | |  | |
| --- | --- | --- | --- | --- | --- | --- | --- | --- |
|  | loss | | LumB | | PR low | | Overall | |
| Survival time | Survival Proportion | Standard Error | Survival Proportion | Standard Error | Survival Proportion | Standard Error | Survival Proportion | Standard Error |
| 0 | 0.917 | 0.0564 | 0.986 | 0.00975 | - | - | 0.978 | 0.0108 |
| 1 | - | - | - | - | 0.938 | 0.0605 | 0.973 | 0.0120 |
| 3 | - | - | - | - | 0.875 | 0.0827 | 0.967 | 0.0131 |
| 6 | - | - | 0.979 | 0.0119 | - | - | 0.962 | 0.0141 |
| 8 | - | - | - | - | 0.813 | 0.0976 | 0.956 | 0.0150 |
| 9 | 0.873 | 0.0686 | 0.972 | 0.0137 | - | - | 0.945 | 0.0168 |
| 10 | 0.829 | 0.0778 | - | - | - | - | 0.940 | 0.0176 |
| 11 | 0.783 | 0.0860 | - | - | - | - | 0.934 | 0.0183 |
| 12 | - | - | 0.965 | 0.0153 | - | - | 0.929 | 0.0190 |
| 13 | 0.734 | 0.0936 | 0.958 | 0.0168 | - | - | 0.917 | 0.0204 |
| 14 | - | - | - | - | - | - | - | - |
| 15 | - | - | - | - | - | - | - | - |
| 16 | - | - | - | - | - | - | - | - |
| 17 | - | - | - | - | - | - | - | - |
| 18 | - | - | 0.950 | 0.0183 | - | - | 0.912 | 0.0211 |
| 19 | - | - | 0.943 | 0.0196 | - | - | 0.906 | 0.0218 |
| 20 | - | - | - | - | 0.745 | 0.110 | 0.900 | 0.0225 |
| 21 | - | - | - | - | - | - | - | - |
| 22 | - | - | - | - | - | - | - | - |
| 24 | - | - | 0.927 | 0.0224 | - | - | 0.887 | 0.0239 |
| 25 | - | - | - | - | - | - | - | - |
| 26 | - | - | - | - | - | - | - | - |
| 27 | 0.685 | 0.0993 | - | - | - | - | 0.881 | 0.0246 |
| 28 | - | - | - | - | - | - | - | - |
| 29 | - | - | - | - | - | - | - | - |
| 30 | - | - | - | - | - | - | - | - |
| 31 | - | - | - | - | - | - | - | - |
| 32 | - | - | - | - | - | - | - | - |
| 33 | - | - | 0.918 | 0.0238 | - | - | 0.874 | 0.0253 |
| 34 | - | - | 0.909 | 0.0253 | - | - | 0.867 | 0.0260 |
| 36 | - | - | - | - | - | - | - | - |
| 37 | 0.636 | 0.104 | - | - | - | - | 0.860 | 0.0268 |
| 38 | - | - | - | - | - | - | - | - |
| 39 | - | - | - | - | - | - | - | - |
| 40 | - | - | - | - | - | - | - | - |
| 41 | 0.587 | 0.107 | 0.898 | 0.0270 | - | - | 0.844 | 0.0284 |
| 42 | - | - | - | - | - | - | - | - |
| 43 | - | - | - | - | - | - | - | - |
| 45 | - | - | 0.888 | 0.0287 | - | - | 0.836 | 0.0293 |
| 46 | - | - | 0.856 | 0.0331 | - | - | 0.812 | 0.0316 |
| 48 | - | - | - | - | - | - | - | - |
| 50 | - | - | 0.845 | 0.0345 | - | - | 0.803 | 0.0324 |
| 52 | - | - | 0.834 | 0.0357 | - | - | 0.795 | 0.0331 |
| 53 | - | - | - | - | - | - | - | - |
| 54 | - | - | - | - | - | - | - | - |
| 55 | - | - | - | - | - | - | - | - |
| 56 | - | - | - | - | - | - | - | - |
| 57 | - | - | - | - | - | - | - | - |
| 58 | - | - | - | - | - | - | - | - |
| 59 | - | - | - | - | - | - | - | - |
| 62 | - | - | - | - | - | - | - | - |
| 63 | - | - | - | - | - | - | - | - |
| 64 | - | - | - | - | - | - | - | - |
| 66 | - | - | 0.820 | 0.0377 | - | - | 0.785 | 0.0342 |
| 67 | - | - | - | - | - | - | - | - |
| 68 | - | - | - | - | - | - | - | - |
| 69 | - | - | - | - | - | - | - | - |
| 72 | - | - | - | - | - | - | - | - |
| 74 | - | - | - | - | - | - | - | - |
| 76 | - | - | - | - | - | - | - | - |
| 78 | - | - | 0.803 | 0.0406 | - | - | 0.772 | 0.0360 |
| 79 | - | - | - | - | - | - | - | - |
| 80 | - | - | - | - | - | - | - | - |
| 81 | - | - | - | - | - | - | - | - |
| 83 | - | - | - | - | - | - | - | - |
| 84 | - | - | - | - | - | - | - | - |
| 85 | - | - | - | - | - | - | - | - |
| 86 | - | - | - | - | - | - | - | - |
| 87 | - | - | - | - | - | - | - | - |
| 88 | - | - | - | - | - | - | - | - |
| 89 | - | - | - | - | - | - | - | - |
| 91 | - | - | - | - | - | - | - | - |
| 92 | - | - | - | - | - | - | - | - |
| 94 | - | - | - | - | - | - | - | - |
| 95 | - | - | 0.777 | 0.0468 | - | - | 0.752 | 0.0404 |
| 96 | - | - | - | - | - | - | - | - |
| 97 | - | - | - | - | - | - | - | - |
| 98 | - | - | - | - | - | - | - | - |
| 99 | - | - | - | - | - | - | - | - |
| 100 | - | - | - | - | - | - | - | - |
| 102 | - | - | - | - | - | - | - | - |
| 105 | - | - | - | - | - | - | - | - |
| 106 | - | - | - | - | - | - | - | - |
| 109 | - | - | - | - | - | - | - | - |
| 110 | - | - | - | - | - | - | - | - |
| 113 | - | - | - | - | - | - | - | - |
| 114 | - | - | - | - | - | - | - | - |
| 115 | - | - | - | - | - | - | - | - |
| 116 | - | - | - | - | - | - | - | - |
| 117 | - | - | - | - | - | - | - | - |
| 119 | - | - | - | - | - | - | - | - |
| 120 | - | - | - | - | - | - | - | - |
| 121 | - | - | - | - | - | - | - | - |
| 125 | - | - | - | - | - | - | - | - |
| 127 | - | - | - | - | - | - | - | - |
| 129 | - | - | - | - | - | - | - | - |
| 130 | - | - | - | - | - | - | - | - |
| 132 | - | - | - | - | - | - | - | - |
| 136 | - | - | - | - | - | - | - | - |
| Endpoint: Observed n | 9.0 | | 22.0 | | 4.0 | |  | |
| Expected n | 3.7 | | 28.1 | | 3.2 | |  | |
| Observed/Expected | 2.4118 | | 0.7829 | | 1.2630 | |  | |

## Comparison of survival curves (Logrank test)

| Chi-squared | 9.0501 |
| --- | --- |
| DF | 2 |
| Significance | P = 0.0108 |

## Hazard ratios^a^ with 95% Confidence Interval

| Factor | loss | LumB | PR low |
| --- | --- | --- | --- |
| loss | - | 0.3246 0.1102 to 0.9558 | 0.5237 0.1171 to 2.3410 |
| LumB | 3.0807 1.0463 to 9.0707 | - | 1.6132 0.5048 to 5.1552 |
| PR low | 1.9096 0.4272 to 8.5368 | 0.6199 0.1940 to 1.9808 | - |

^a^ Column/Row


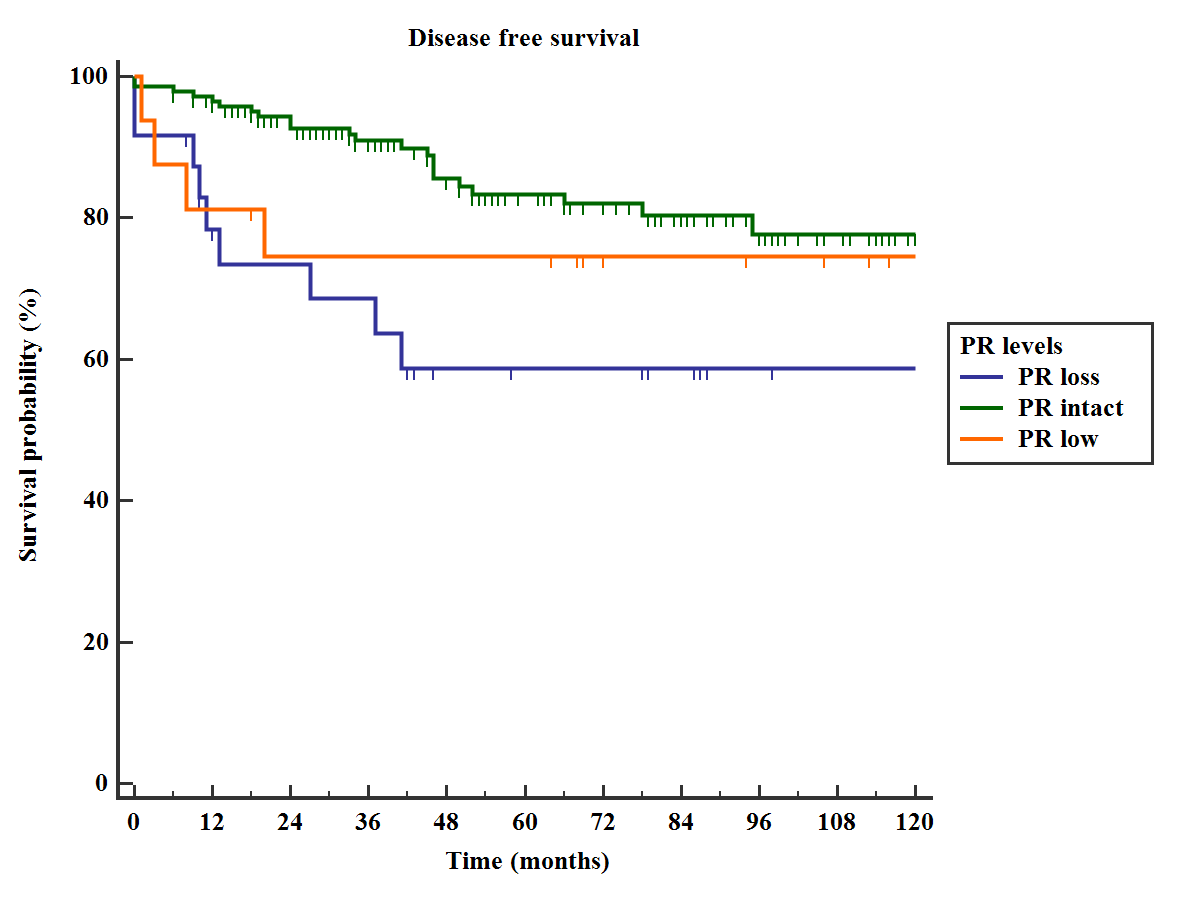


# Kaplan-Meier survival analysis

| Survival time | os |
| --- | --- |
| Endpoint | 사망유무__death__1__live__0 |
| Factor codes | BCL_33 |

## Cases summary

|  | Number of events ^a^ | | Number censored ^b^ | |  |
| --- | --- | --- | --- | --- | --- |
| Factor | N | % | N | % | Total sample size |
| 1 | 11 | 25.00 | 33 | 75.00 | 44 |
| 2 | 10 | 7.14 | 130 | 92.86 | 140 |
| Overall | 21 | 11.41 | 163 | 88.59 | 184 |

^a^ 사망유무__death__1__live__0 = 1
^b^ 사망유무__death__1__live__0 = 0

## Mean and median survival

| Factor | Mean | SE | 95% CI for the mean | Median | 95% CI for the median |
| --- | --- | --- | --- | --- | --- |
| 1 | 99.614 | 7.840 | 84.247 to 114.981 | - | - |
| 2 | 126.773 | 2.784 | 121.316 to 132.229 | - | - |
| Overall | 121.124 | 3.026 | 115.192 to 127.055 | - | - |

## Survival table [[Show]](javascript:showdiv('d10','d11','table1');)

## Survival table [[Hide]](javascript:hidediv('d10','d11','table1');)

|  | Factor | | | |  | |
| --- | --- | --- | --- | --- | --- | --- |
|  | 1 | | 2 | | Overall | |
| Survival time | Survival Proportion | Standard Error | Survival Proportion | Standard Error | Survival Proportion | Standard Error |
| 6 | - | - | - | - | - | - |
| 7 | 0.977 | 0.0225 | - | - | 0.995 | 0.00545 |
| 8 | - | - | - | - | - | - |
| 9 | - | - | - | - | - | - |
| 10 | 0.955 | 0.0314 | - | - | 0.989 | 0.00773 |
| 11 | 0.932 | 0.0380 | 0.993 | 0.00733 | 0.978 | 0.0109 |
| 12 | 0.909 | 0.0433 | - | - | 0.972 | 0.0122 |
| 14 | 0.886 | 0.0481 | - | - | 0.967 | 0.0134 |
| 15 | 0.862 | 0.0522 | - | - | 0.961 | 0.0145 |
| 16 | - | - | - | - | - | - |
| 17 | - | - | - | - | - | - |
| 18 | - | - | - | - | - | - |
| 19 | - | - | 0.985 | 0.0107 | 0.955 | 0.0155 |
| 20 | - | - | - | - | - | - |
| 21 | - | - | - | - | - | - |
| 22 | - | - | - | - | - | - |
| 24 | 0.836 | 0.0568 | - | - | 0.949 | 0.0166 |
| 25 | - | - | - | - | - | - |
| 26 | - | - | - | - | - | - |
| 27 | 0.809 | 0.0610 | - | - | 0.942 | 0.0177 |
| 28 | - | - | - | - | - | - |
| 29 | - | - | - | - | - | - |
| 30 | - | - | - | - | - | - |
| 31 | - | - | - | - | - | - |
| 32 | - | - | - | - | - | - |
| 33 | 0.780 | 0.0653 | - | - | 0.936 | 0.0188 |
| 34 | - | - | - | - | - | - |
| 36 | - | - | 0.976 | 0.0139 | 0.929 | 0.0199 |
| 37 | 0.749 | 0.0698 | - | - | 0.922 | 0.0210 |
| 38 | - | - | - | - | - | - |
| 39 | - | - | - | - | - | - |
| 40 | - | - | - | - | - | - |
| 41 | - | - | 0.966 | 0.0168 | 0.914 | 0.0221 |
| 42 | - | - | - | - | - | - |
| 43 | - | - | 0.956 | 0.0192 | 0.907 | 0.0232 |
| 44 | - | - | 0.946 | 0.0215 | 0.899 | 0.0243 |
| 45 | - | - | 0.936 | 0.0235 | 0.891 | 0.0253 |
| 46 | - | - | - | - | - | - |
| 48 | - | - | - | - | - | - |
| 49 | - | - | 0.926 | 0.0254 | 0.883 | 0.0264 |
| 50 | - | - | - | - | - | - |
| 51 | - | - | - | - | - | - |
| 52 | - | - | - | - | - | - |
| 53 | - | - | - | - | - | - |
| 54 | - | - | 0.915 | 0.0274 | 0.874 | 0.0275 |
| 55 | - | - | - | - | - | - |
| 56 | - | - | - | - | - | - |
| 57 | - | - | - | - | - | - |
| 58 | - | - | - | - | - | - |
| 59 | - | - | - | - | - | - |
| 61 | - | - | - | - | - | - |
| 62 | - | - | - | - | - | - |
| 63 | - | - | - | - | - | - |
| 64 | - | - | - | - | - | - |
| 66 | - | - | - | - | - | - |
| 67 | - | - | - | - | - | - |
| 68 | - | - | - | - | - | - |
| 69 | - | - | - | - | - | - |
| 71 | - | - | - | - | - | - |
| 72 | - | - | - | - | - | - |
| 74 | - | - | - | - | - | - |
| 76 | - | - | - | - | - | - |
| 77 | 0.696 | 0.0828 | - | - | 0.861 | 0.0301 |
| 78 | - | - | - | - | - | - |
| 79 | - | - | 0.897 | 0.0322 | 0.847 | 0.0326 |
| 80 | - | - | - | - | - | - |
| 81 | - | - | - | - | - | - |
| 83 | - | - | - | - | - | - |
| 84 | - | - | - | - | - | - |
| 85 | - | - | - | - | - | - |
| 86 | - | - | - | - | - | - |
| 87 | - | - | - | - | - | - |
| 88 | - | - | - | - | - | - |
| 89 | - | - | - | - | - | - |
| 91 | - | - | - | - | - | - |
| 92 | - | - | - | - | - | - |
| 94 | - | - | - | - | - | - |
| 96 | - | - | - | - | - | - |
| 97 | - | - | - | - | - | - |
| 98 | - | - | - | - | - | - |
| 99 | - | - | - | - | - | - |
| 100 | - | - | - | - | - | - |
| 102 | - | - | - | - | - | - |
| 105 | - | - | - | - | - | - |
| 106 | - | - | - | - | - | - |
| 109 | - | - | - | - | - | - |
| 110 | - | - | - | - | - | - |
| 113 | - | - | - | - | - | - |
| 114 | - | - | - | - | - | - |
| 115 | - | - | - | - | - | - |
| 116 | - | - | - | - | - | - |
| 117 | - | - | - | - | - | - |
| 119 | - | - | - | - | - | - |
| 120 | - | - | - | - | - | - |
| 121 | - | - | - | - | - | - |
| 122 | - | - | - | - | - | - |
| 125 | - | - | - | - | - | - |
| 127 | - | - | - | - | - | - |
| 129 | - | - | - | - | - | - |
| 130 | - | - | - | - | - | - |
| 132 | - | - | - | - | - | - |
| 136 | - | - | - | - | - | - |
| Endpoint: Observed n | 11.0 | | 10.0 | |  | |
| Expected n | 4.3 | | 16.7 | |  | |
| Observed/Expected | 2.5434 | | 0.5997 | |  | |

## Comparison of survival curves (Logrank test)

| Chi-squared | 13.0263 |
| --- | --- |
| DF | 1 |
| Significance | P = 0.0003 |

## Hazard ratios^a^ with 95% Confidence Interval

| Factor | 1 | 2 |
| --- | --- | --- |
| 1 | - | 0.2358 0.08188 to 0.6790 |
| 2 | 4.2411 1.4728 to 12.2125 | - |

^a^ Column/Row


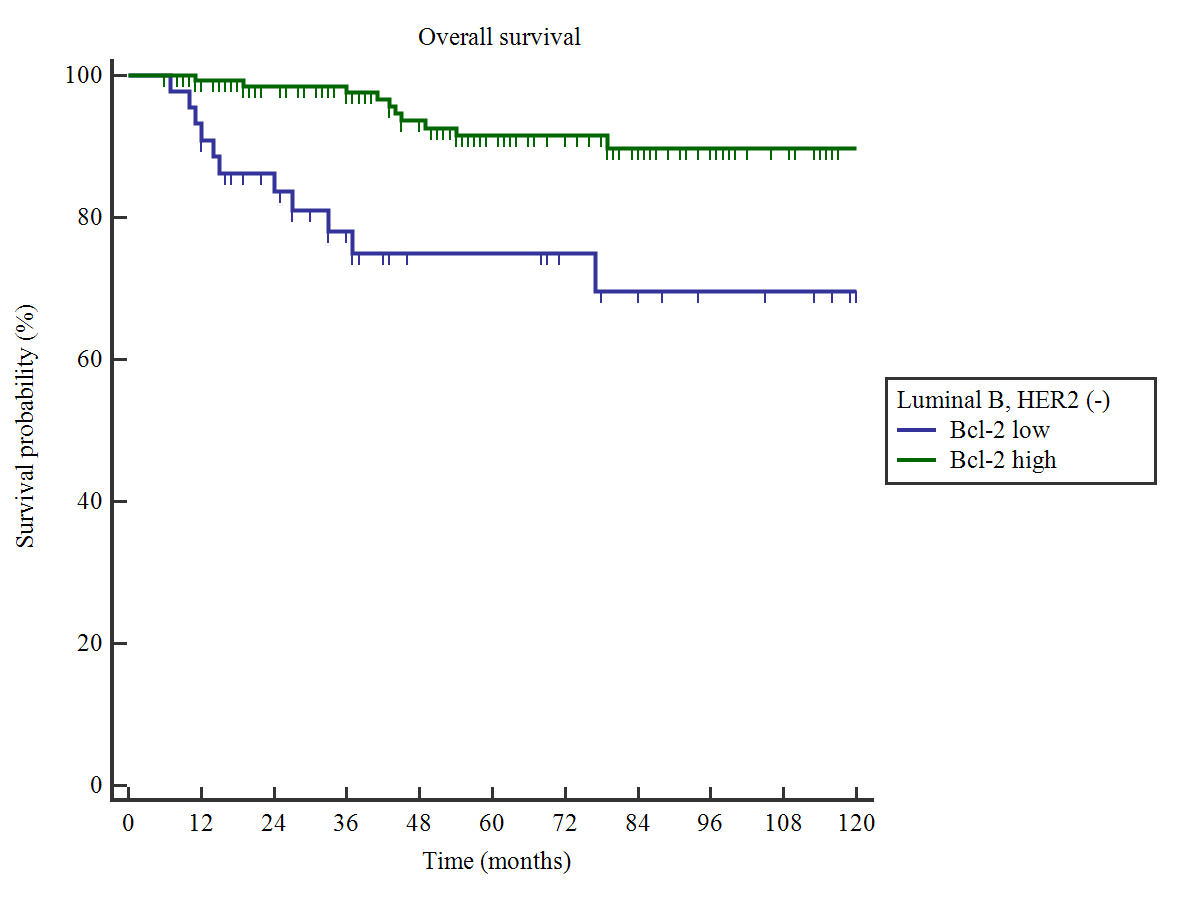


# Kaplan-Meier survival analysis

| Survival time | dfs |
| --- | --- |
| Endpoint | 재발_유무 |
| Factor codes | BCL_33 |

## Cases summary

|  | Number of events ^a^ | | Number censored ^b^ | |  |
| --- | --- | --- | --- | --- | --- |
| Factor | N | % | N | % | Total sample size |
| 1 | 14 | 31.82 | 30 | 68.18 | 44 |
| 2 | 21 | 15.00 | 119 | 85.00 | 140 |
| Overall | 35 | 19.02 | 149 | 80.98 | 184 |

^a^ 재발_유무 = 1
^b^ 재발_유무 = 0

## Mean and median survival

| Factor | Mean | SE | 95% CI for the mean | Median | 95% CI for the median |
| --- | --- | --- | --- | --- | --- |
| 1 | 89.917 | 8.686 | 72.892 to 106.942 | - | - |
| 2 | 116.044 | 3.965 | 108.272 to 123.815 | - | - |
| Overall | 110.865 | 3.775 | 103.466 to 118.263 | - | - |

## Survival table [[Show]](javascript:showdiv('d12','d13','table1');)

## Survival table [[Hide]](javascript:hidediv('d12','d13','table1');)

|  | Factor | | | |  | |
| --- | --- | --- | --- | --- | --- | --- |
|  | 1 | | 2 | | Overall | |
| Survival time | Survival Proportion | Standard Error | Survival Proportion | Standard Error | Survival Proportion | Standard Error |
| 0 | 0.977 | 0.0225 | 0.979 | 0.0122 | 0.978 | 0.0108 |
| 1 | - | - | 0.971 | 0.0141 | 0.973 | 0.0120 |
| 3 | - | - | 0.964 | 0.0157 | 0.967 | 0.0131 |
| 6 | 0.955 | 0.0314 | - | - | 0.962 | 0.0141 |
| 8 | 0.932 | 0.0380 | - | - | 0.956 | 0.0150 |
| 9 | 0.886 | 0.0478 | - | - | 0.945 | 0.0168 |
| 10 | 0.864 | 0.0517 | - | - | 0.940 | 0.0176 |
| 11 | 0.841 | 0.0551 | - | - | 0.934 | 0.0183 |
| 12 | - | - | 0.957 | 0.0172 | 0.929 | 0.0190 |
| 13 | 0.818 | 0.0583 | 0.949 | 0.0186 | 0.917 | 0.0204 |
| 14 | - | - | - | - | - | - |
| 15 | - | - | - | - | - | - |
| 16 | - | - | - | - | - | - |
| 17 | - | - | - | - | - | - |
| 18 | - | - | 0.942 | 0.0200 | 0.912 | 0.0211 |
| 19 | 0.793 | 0.0616 | - | - | 0.906 | 0.0218 |
| 20 | - | - | 0.934 | 0.0214 | 0.900 | 0.0225 |
| 21 | - | - | - | - | - | - |
| 22 | - | - | - | - | - | - |
| 24 | - | - | 0.917 | 0.0240 | 0.887 | 0.0239 |
| 25 | - | - | - | - | - | - |
| 26 | - | - | - | - | - | - |
| 27 | - | - | 0.909 | 0.0252 | 0.881 | 0.0246 |
| 28 | - | - | - | - | - | - |
| 29 | - | - | - | - | - | - |
| 30 | - | - | - | - | - | - |
| 31 | - | - | - | - | - | - |
| 32 | - | - | - | - | - | - |
| 33 | 0.763 | 0.0660 | - | - | 0.874 | 0.0253 |
| 34 | - | - | 0.900 | 0.0265 | 0.867 | 0.0260 |
| 36 | - | - | - | - | - | - |
| 37 | 0.732 | 0.0705 | - | - | 0.860 | 0.0268 |
| 38 | - | - | - | - | - | - |
| 39 | - | - | - | - | - | - |
| 40 | - | - | - | - | - | - |
| 41 | 0.662 | 0.0791 | - | - | 0.844 | 0.0284 |
| 42 | - | - | - | - | - | - |
| 43 | - | - | - | - | - | - |
| 45 | - | - | 0.889 | 0.0282 | 0.836 | 0.0293 |
| 46 | - | - | 0.858 | 0.0325 | 0.812 | 0.0316 |
| 48 | - | - | - | - | - | - |
| 50 | 0.623 | 0.0835 | - | - | 0.803 | 0.0324 |
| 52 | - | - | 0.847 | 0.0338 | 0.795 | 0.0331 |
| 53 | - | - | - | - | - | - |
| 54 | - | - | - | - | - | - |
| 55 | - | - | - | - | - | - |
| 56 | - | - | - | - | - | - |
| 57 | - | - | - | - | - | - |
| 58 | - | - | - | - | - | - |
| 59 | - | - | - | - | - | - |
| 62 | - | - | - | - | - | - |
| 63 | - | - | - | - | - | - |
| 64 | - | - | - | - | - | - |
| 66 | - | - | 0.833 | 0.0360 | 0.785 | 0.0342 |
| 67 | - | - | - | - | - | - |
| 68 | - | - | - | - | - | - |
| 69 | - | - | - | - | - | - |
| 72 | - | - | - | - | - | - |
| 74 | - | - | - | - | - | - |
| 76 | - | - | - | - | - | - |
| 78 | - | - | 0.816 | 0.0392 | 0.772 | 0.0360 |
| 79 | - | - | - | - | - | - |
| 80 | - | - | - | - | - | - |
| 81 | - | - | - | - | - | - |
| 83 | - | - | - | - | - | - |
| 84 | - | - | - | - | - | - |
| 85 | - | - | - | - | - | - |
| 86 | - | - | - | - | - | - |
| 87 | - | - | - | - | - | - |
| 88 | - | - | - | - | - | - |
| 89 | - | - | - | - | - | - |
| 91 | - | - | - | - | - | - |
| 92 | - | - | - | - | - | - |
| 94 | - | - | - | - | - | - |
| 95 | - | - | 0.789 | 0.0464 | 0.752 | 0.0404 |
| 96 | - | - | - | - | - | - |
| 97 | - | - | - | - | - | - |
| 98 | - | - | - | - | - | - |
| 99 | - | - | - | - | - | - |
| 100 | - | - | - | - | - | - |
| 102 | - | - | - | - | - | - |
| 105 | - | - | - | - | - | - |
| 106 | - | - | - | - | - | - |
| 109 | - | - | - | - | - | - |
| 110 | - | - | - | - | - | - |
| 113 | - | - | - | - | - | - |
| 114 | - | - | - | - | - | - |
| 115 | - | - | - | - | - | - |
| 116 | - | - | - | - | - | - |
| 117 | - | - | - | - | - | - |
| 119 | - | - | - | - | - | - |
| 120 | - | - | - | - | - | - |
| 121 | - | - | - | - | - | - |
| 125 | - | - | - | - | - | - |
| 127 | - | - | - | - | - | - |
| 129 | - | - | - | - | - | - |
| 130 | - | - | - | - | - | - |
| 132 | - | - | - | - | - | - |
| 136 | - | - | - | - | - | - |
| Endpoint: Observed n | 14.0 | | 21.0 | |  | |
| Expected n | 7.4 | | 27.6 | |  | |
| Observed/Expected | 1.8873 | | 0.7614 | |  | |

## Comparison of survival curves (Logrank test)

| Chi-squared | 7.4719 |
| --- | --- |
| DF | 1 |
| Significance | P = 0.0063 |

## Hazard ratios^a^ with 95% Confidence Interval

| Factor | 1 | 2 |
| --- | --- | --- |
| 1 | - | 0.4034 0.1794 to 0.9075 |
| 2 | 2.4788 1.1020 to 5.5756 | - |

^a^ Column/Row


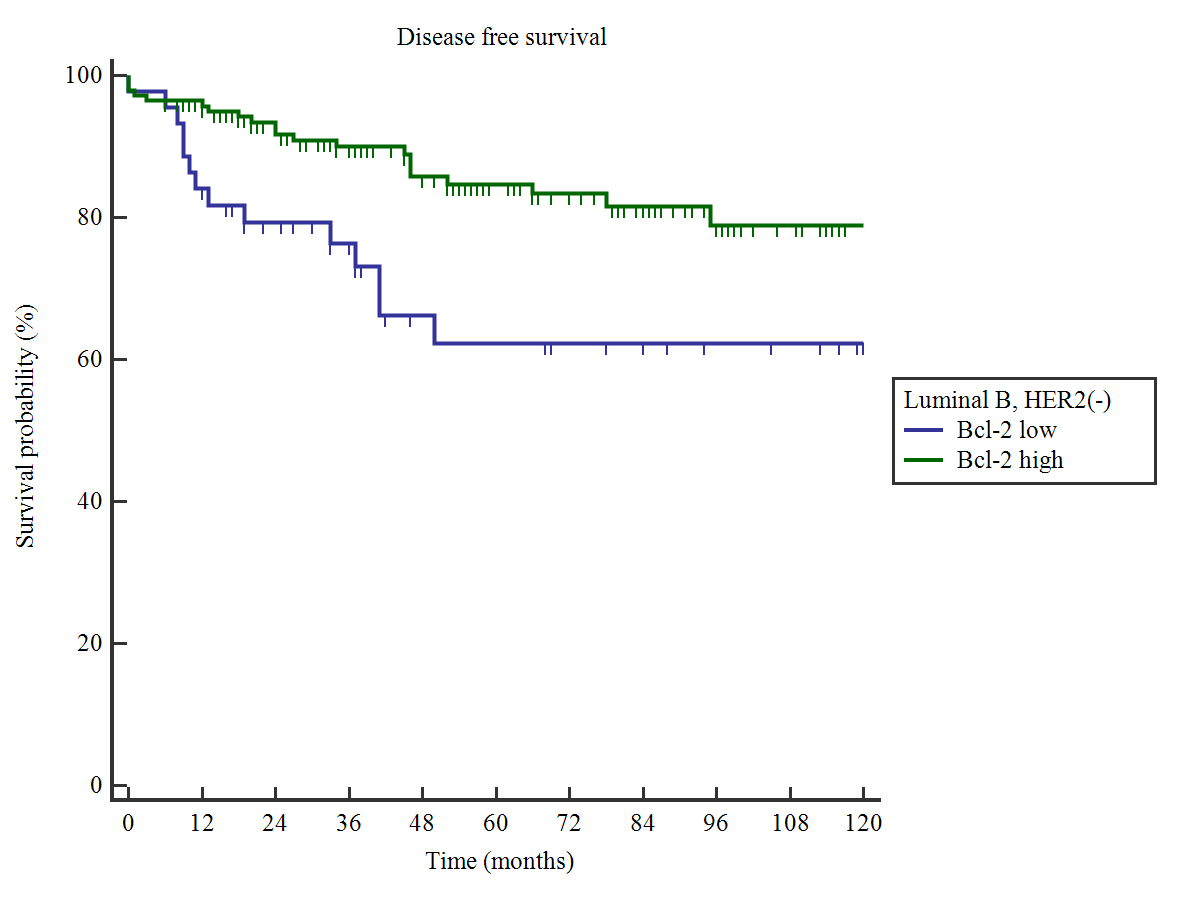

Supplement: Additional file 1: — Result of statistic analysis. (DOCX 389 kb) [file 12957_2016_999_MOESM1_ESM.docx]
